# Obesity, Physical Activity and Sedentary Behavior Amongst British and Saudi Youth: A Cross-Cultural Study

**DOI:** 10.3390/ijerph9041490

**Published:** 2012-04-16

**Authors:** Yahya Al-Nakeeb, Mark Lyons, Peter Collins, Anwar Al-Nuaim, Hazzaa Al-Hazzaa, Michael J. Duncan, Alan Nevill

**Affiliations:** 1 School of Human Sciences, Newman University College, Genners Lane, Bartley Green, Birmingham, B32 3NT, UK; Email: mark.lyons@ul.ie (M.L.); p.collins@newman.ac.uk (P.C.); NUAI200@newman.ac.uk (A.A.-N.); 2 Exercise Physiology Laboratory, King Saud University, P.O. Box 2458, Riyadh 11451, Saudi Arabia; Email: alhazzaa@ksu.edu.sa; 3 Faculty of Health and Life Sciences, Coventry University, James Starley Building, Priory Street, Coventry, CV1 5FB, UK; Email: aa8396@coventry.ac.uk; 4 School of Performing Arts and Leisure, University of Wolverhampton, Walsall Campus, Gorway Road, Walsall, WS1 3BD, UK; Email: a.m.nevill@wlv.ac.uk

**Keywords:** obesity, physical activity, young people, lifestyle, environment

## Abstract

This study explores differences in weight status, obesity and patterns of physical activity (PA) in relation to gender and age of youth from two culturally, environmentally and geographically diverse countries, the United Kingdom (UK) and Saudi Arabia (SA). A total of 2,290 males and females (15–17 years) volunteered to participate in this study. Participants completed a validated self-report questionnaire that contained 47 items relating to patterns of PA, sedentary activity and eating habits. The questionnaire allows the calculation of total energy expenditure in metabolic equivalent (MET-min) values per week. Significant differences in percentage of overweight/obese and levels of PA were evident between the youth from the two countries, with males being generally more physically active than females. Additionally, there were significant associations between Body Mass Index (BMI), PA and sedentary behaviors; the youth with higher BMI reported lower levels of PA and higher amounts of sedentary time. These findings highlight the diverse nature of lifestyle of youth living in different geographical areas of the world and the need for further research to explore the socio-cultural factors that impact on the prevalence of obesity and patterns of PA of youth in different populations.

## 1. Introduction

Recent large-scale epidemiological studies utilizing valid measures of PA have demonstrated stronger associations between PA and health benefits than have been observed previously and helped to clarify dose–response relationships between activity and specific health outcomes [1,2,3]. Physical inactivity and obesity are leading risk factors for global mortality accounting for 6% and 5% respectively [4]. The strength of the relationship between PA and health outcomes persists throughout people’s lives, highlighting the potential health gains that could be achieved if people continue to be active. Recent guidelines on recommended levels of PA for children and young people (5–18 years) indicate that they should engage in moderate to vigorous-intensity PA for at least 60 minutes and up to several hours every day, and they should minimize the amount of time being sedentary [5]. PA provides important health benefits for young people. This conclusion is based upon evidence from observational studies in which higher levels of activity were associated with more favorable health outcomes and experimental studies in which exercise treatments resulted in improvements in health-related measures [6,7].

Although the rise of the global obesity epidemic during the past few decades is substantial, there are wide variations in obesity prevalence across countries and populations due to socioeconomic, cultural and transport differences in national and local environments [8]. The increased mechanization and motorization in the first half of the 20th century were accompanied by corresponding decreases in food energy supply that helped in preserving low obesity prevalence. However, in the 1970s–80s, an energy balance flipping point seems to have occurred in many high-income countries [9]. This was then followed by most middle-income and many low-income countries who have joined the global surge in obesity prevalence in adults and children [10,11]. It appears that the most obvious environmental precondition for a population to develop obesity is sufficient wealth; therefore, a positive relationship seems to exist between personal wealth and BMI with economic prosperity being regarded as an enabler for obesity [12]. Lifestyle changes in high-income countries have led to a decrease in the energy expenditure needed for daily life and an increase in sedentary activities and weight gain. Research findings show very wide variations in obesity prevalence globally, particularly for women [8]. Many of the reasons for the variations across populations are perceptive. For example, Ethiopia does not have sufficient national wealth for obesity to have manifested itself, and populations in Hong Kong and Jordan have had a greater exposure to obesogenic food environments than do their counterparts in China and Yemen. However, many complexities exist in understanding why some populations and subpopulations are more susceptible to the drivers of obesity than others, and how mediating factors affect different population groups [8].

The cross-cultural differences in lifestyle and PA in relation to overweight and obesity are clearly of interest to researchers and policy makers in most countries to explore and identify intervention strategies. Therefore, the focus of the current study is on two very diverse countries in terms of social, cultural and environmental characteristics: United Kingdom (UK) and Saudi Arabia (SA). However, both are regarded as high income countries and have a growing problem of obesity among their child and adult populations. In the UK, with the industrial revolution and more recently the emergence of technological advances, a serious mismatch has emerged between food availability and the energy required to access food. This has, inevitably, led to a new pandemic of metabolic conditions such as obesity and type 2 diabetes. Meanwhile, over the past 2–3 decades, SA has witnessed significant lifestyle changes due to rapid urbanization, dominance of the automobile for personal travel, availability of high-fat and dense-caloric foods, satellite TV, increased reliance on computer and telecommunication technology and decreased occupational work demands [13]. These lifestyle changes have had a considerable impact on reducing the physical requirements of daily life and have encouraged sedentary lifestyles amongst both young people and adults.

According to the World Health Organization [14], the most important risk factors of non-communicable diseases in the Gulf countries included high blood pressure, high concentrations of cholesterol in the blood, inadequate intake of fruit and vegetables, overweight or obesity, physical inactivity and tobacco use. Five of these risks are closely related to inappropriate diet and physical inactivity. Participation in health-enhancing PA is a key determinant of energy expenditure in youth and leads to improved cardiovascular and metabolic fitness as well as bone health [15]. In addition, recent research findings have shown that TV viewing (sedentary activity) and PA appear to be separate entities and are independently associated with metabolic risk [16]. Sedentary behavior is not simply a lack of PA but is a cluster of individual behaviors where sitting or lying is the dominant mode of posture and energy expenditure is very low; it is a multi-faceted behavior that might take place at work, school or home [17]. Childhood and adolescence are crucial stages of development where lifestyle habits are formed and set. The behavior patterns that are established during early years have immediate and long-term implications on health and well-being. Among the multitude of social, emotional and institutional transitions that take place are reductions in habitual levels of PA and increased participation in certain sedentary behaviors which have significant implications to public health [18]. Young people’s PA and health habits are normally influenced by a number of physical and social environmental determinants. These include: culture, societal norms, attitudes, built environment and climate.

In the UK, levels of PA and sedentary behavior among young people tend to promote obesity [19]. The excess weight gain which has taken place by the time of adolescence may often have occurred largely by the time children enter primary school [20]. Prevalence of overweight and obesity have been increasing in the UK with almost one in four children either overweight or obese [21]. Likewise, in SA, an alarming level of physical inactivity among the Saudi populations has been reported, predisposing them to health problems [22,23]. Although childhood obesity has been observed and widely reported in developed countries; in recent years, there is an ever-increasing prevalence of obesity in developing countries. For example, it has been reported that the prevalence of childhood obesity is high in the Middle East, Central and Eastern Europe [24]; and, in Saudi Arabia, one in every six children aged 6–18 years is obese [25].

The major aims of this study were: (1) to explore the lifestyle of youth from culturally and environmentally diverse cities, two from Central England (Birmingham & Coventry) and one from the Eastern Province of Saudi Arabia (Al-Ahsa); (2) to examine differences in patterns of PA and weight status in relation to gender, age and geographical location. It is expected that the findings from this cross-cultural study will provide substantive information on youth PA and inactivity patterns as well as the prevalence of obesity amongst young males and females in the three cities. Additionally, the study will afford a baseline indication of the prevalence of obesity and patterns of PA and inactivity and contribute towards addressing the limited available comparative research data on youth across different cultures.

## 2. Methods

### 2.1. Locations

The study was carried out in three cities that are culturally and environmentally diverse; two from Central England (Birmingham and Coventry) and one from the Eastern Province of Saudi Arabia (Al-Ahsa). Birmingham is a large metropolitan city in the heart of England. It is the second largest city in England and the most populous British city outside of London, with an estimated population of 1,036,900 [26]. Coventry is also situated in Central England and is the second largest city in the Midlands with a population of approximately 300,848. Coventry city center underwent major development during the 1950’s and 1960’s after being decimated during the Second World War [27]. Manufacturing has traditionally dominated the economy in both Midland cities although over the past decade, the downturn in manufacturing trade has coincided with the emergence of a strong service sector accounting for the vast majority of employment in Birmingham. In both Coventry and Birmingham there is a large population of ethnic minority groups [26,27]. Al-Ahsa is the largest governorate in the Eastern Province of Saudi Arabia, and comprises several urban settlements in addition to both rural and desert communities. It has the second highest population in Saudi Arabia of approximately 908,000 people and has the longest history of adopting a Western lifestyle with respect to nutritional habits. Its economy is driven by agriculture and oil production, with some of the richest oil fields in the world found in this region [28]. 

### 2.2. Participants

A total of 2,290 young males (1,185) and females (1,105) aged 15–17 years-old from the cities of Al-Ahsa (KSA), Birmingham and Coventry (UK) volunteered to take part in this study following informed consent from participants and parents along with institutional ethical approval. A stratified sample representing the different geographical areas in each city was identified; the sample size was determined so that it would be within ± 0.05 of the population proportion with a 95% confidence level. Participants were randomly selected from secondary schools in each respective city. In Al-Ahsa, 1,138 youth took part, with 538 female (*M* = 16.51 ± 0.68 yrs) and 600 males (*M* = 16.60 ± 0.60 yrs). In Birmingham, a total of 637 participants took part, with 329 females (*M* = 15.55 ± 0.74 yrs) and 308 males (*M* = 15.58 ± 0.75 yrs). Similarly, in Coventry, a total of 515 participants took part, with 238 females (*M* = 15.22 ± 0.46 yrs) and 277 males (*M* = 15.32 ± 0.57 yrs).

### 2.3. Lifestyle Questionnaire

A validated self-report questionnaire was used to assess the PA patterns, sedentary activity and dietary habits of the selected sample. The Arabic version of this research tool was previously used in the Arab Teens Lifestyle Study (ATLS) [13] and contained 47 items relating to patterns of PA, sedentary behaviors and eating habits. Due to the significant amount of data concerning eating habits, this aspect will be the topic of a future article. Hence, the focus of the current article is young people’s PA and sedentary behaviors. The PA questionnaire was shown to have a high reliability (ICC = 0.85; 95% CL = 0.70–0.93) and an acceptable validity (r = 0.30; *p* < 0.05) against pedometers on a sample of males 15–25 year-old [29]. In another validity study involving both males and females aged 14–19 years it was found to have a validity coefficient of 0.37 (*p* < 0.05) against pedometers [30]. The adaptation of the questionnaire from Arabic to English language applied the translation/back translation method [31] using an expert committee. The translated questionnaire did not require any changes in either the number or meaning of the questions. A pilot study with a sample of British youth 15–18 years to assess the cross-cultural suitability of the questionnaire demonstrated its appropriateness and the full understanding of questions content by the youth. No modifications were required, thus construct validity was assumed.

This questionnaire covers all domains of PA including transport, household, fitness and sports, representing light-, moderate- and vigorous-intensity physical activities. Moderate-intensity PA includes activities such as normal pace walking, brisk walking, recreational swimming, household activities and recreational sports such as volleyball, badminton and table tennis. Vigorous-intensity PA included activities such as stair climbing, jogging, running, cycling, self-defense, weight training and sports such as soccer, basketball, handball and singles tennis. Physical activities were assigned MET values based on the compendium of PA [32] and the compendium of PA for youth [33]. Moderate-intensity recreational sports were assigned an average MET value equivalent to 4 METs. Vigorous-intensity sports were assigned an average MET value equivalent to 8 METs. Slow walking, normal pace walking, and brisk walking were assigned MET values of 2.8, 3.5 and 4.5 METs respectively, based on modified METs values from the compendium of PA for youth [33,34]. The questionnaire allows the calculation of total energy expenditure per week based on metabolic equivalent (MET-min) values of all types of physical activities reported by the participant. To measure the participants’ levels of PA, the total METs-min per week and the METs-min per week spent in vigorous- and moderate-intensity PA were used. The classifications adopted for activity levels in this paper were based on two cut-off points of 30 minutes and 60 minutes per day of at least a moderate level of PA. This was then converted into three activity categories based on total METs minute per week as follows: Active: > 1680 METs-min per week (60 minutes × 7 days × 4 METs). This daily 60 minutes of at least a moderate level of PA is based on recent PA recommendations [4]. Minimally active: ≥ 840 < 1680 (30 minutes × 7 days × 4 METs). Inactive: < 840 METs-min per week [6].

### 2.4. BMI Measurement

Body weight was measured to the nearest 100 grams using Seca weight scales (Seca Ltd., Hamburg, Germany). Participants were weighed barefooted and without excess outer clothing. To ensure measurement accuracy, the scale was checked for a zero reading before each weighing. Height was measured to the nearest 0.5 centimeter using a Seca portable height measure (Seca Ltd., Hamburg, Germany). BMI was calculated using the formulae: Weight (kg)/Height (m^2^). BMI was classified according to the International Obesity Task Force (IOTF) criteria [35]. 

### 2.5. Statistical Analysis

A range of statistical procedures were performed on the data to establish associations and differences in patterns of PA and overweight/obesity of young people between the three cities. PA levels and BMI of youth from the different cities were analyzed according to gender and age-group using 2-way and 3-way analyses of variance (ANOVA). Chi-square analysis was conducted on the frequency data to examine differences on the PA index between youth from the three cities. Descriptive statistics were utilized to highlight the prevalence of overweight and obesity as well as classifications according to activity index. Furthermore, Pearson’s correlations were performed to establish relationships between health status variables (e.g., BMI and PA levels) and sedentary lifestyle habits such as TV viewing time and computer usage. SPSS version 19 was used for all analyses [36].

## 3. Results

The descriptive characteristics of the main dependent variables in males and females across the three cities are presented in Table 1. These include: levels of PA, anthropometric and obesity measures for participants from the three different cities. A wide range of differences in PA, sedentary time and obesity measures were evident between youth from the three cities as well as between males and females. 

**Table 1:** Mean (± SD) of the main dependent variables across the three cities.

Variable	Al-Ahsa		Birmingham		Coventry	
	Male	Female	Male	Female	Male	Female
N	600	538	308	329	277	238
Age (yrs)	16.60 ± 0.60	16.51 ± 0.67	15.59 ± 0.75	15.55 ± 0.74	15.32 ± 0.57	15.22 ± 0.46
Weight (kg)	67.46 ± 20.49	58.02 ± 17.61	65.68 ± 12.71	59.09 ± 11.98	62.96 ± 15.09	55.55 ± 10.32
Height (cm)	167.68 ± 6.81	154.89 ± 5.42	171.52 ± 7.97	161.93 ± 6.96	171.77 ± 8.53	161.08 ± 7.04
BMI (kg/m^2^)	23.90 ± 6.75	24.14 ± 7.09	22.26 ± 3.70	22.50 ± 4.12	21.38± 4.89	21.46 ± 3.38
Time spent on TV (hrs/day)	2.51 ± 1.81	2.61 ± 1.91	2.67 ± 1.73	2.81 ± 1.72	2.72 ± 1.72	2.88 ± 1.63
Time spent on computer (hrs/day)	2.48 ± 2.07	3.18 ± 2.40	2.55 ± 1.70	2.84 ± 1.73	2.79 ± 1.88	2.95 ± 1.88
Sedentary activity (hrs/day) (TV+computer)	4.99 ± 3.02	5.78 ± 2.57	5.22 ± 2.82	5.65 ± 2.66	5.50 ± 2.74	5.87 ± 2.81
Total METs-min/week (vigorous)	1759 ± 2042	129 ± 244	3268 ± 2547	1172 ± 1315	3531 ± 2691	1488 ± 1610
Total METs-min/week (moderate)	692 ± 782	570 ± 503	1478 ± 1010	1452 ± 1053	1309 ± 919	1488 ± 1020
Total METs-min/week	2337 ± 2323	565 ± 630	4710 ± 2950	2617 ± 1883	4804 ± 3048	2968 ±2 178

### 3.1. Differences in Physical Activity Levels

The one-way ANOVA revealed that there were highly significant differences in the levels of PA (as measured by total METs-min) of youth across the three cities (F_2,2244 _= 258.02, *p* < 0.001). Bonferroni *post-hoc* analyses revealed that youth from Al-Ahsa were significantly (*p* < 0.001) less active than youth in both Birmingham and Coventry (1,495, 3,627 and 3,949 METs/wk respectively). A two-way ANOVA was conducted to test for gender, city and their interactions. The analysis confirms the significant differences between cities (*p* < 0.001) and genders (*p* < 0.001). However, there was no significant interaction between gender and city with regards to either total PA or walking PA (*p* > 0.05).

Chi-square test of independence revealed a significant difference in the PA levels between youth in Birmingham, Coventry and Al-Ahsa (χ^2^_4_ = 653.85, *p* < 0.001). The percentages of ‘active’ youth (males and females combined) in Birmingham, Coventry and Al-Ahsa were 72.3%, 77.2% and 26%, respectively (Table 2).

**Table 2:** Levels of physical activity of males and females across the three cities.

City	Gender	N	Physical Activity Index (METs-min/week)		
			Inactive	Minimally Active	Active
**Birmingham**	Males	308	6.5%	11.7%	81.8%
	Females	328	13.7%	22.9%	63.4%
	Total	636	10.2%	17.5%	72.3%
**Coventry**	Males	277	6.5%	10.1%	83.4%
	Females	237	11%	19%	70%
	Total	514	8.6%	14.2%	77.2%
**Al-Ahsa**	Males	576	34.5%	19.6%	45.8%
	Females	531	81.4%	14.1%	4.5%
	Total	1,107	57 %	17%	26%

**Figure 1:**
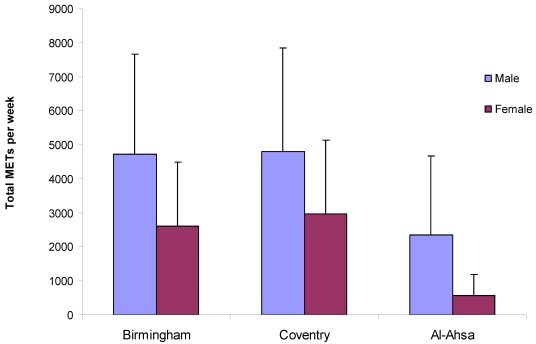
Total METs-min per week of males and females across the three cities.

With respect to gender, univariate ANOVA revealed significant differences between males and females across each of the three cities (*p* < 0.001 for all analyses) with males from Birmingham and Coventry being more physically active than males from Al-Ahsa (Figure 1). However, no significant differences were evident between males from Birmingham and Coventry (*p* > 0.05). As for females, those from Al-Ahsa were less physically active than those from Birmingham or Coventry (*p* < 0.001). Additionally, there were significant differences between females from Coventry and those from Birmingham (*p* = 0.020), with females from Coventry reporting higher levels of PA than females from Birmingham (2967 and 2616 METs per week, respectively). These differences are illustrated in Figure 1.

**Figure 2:**
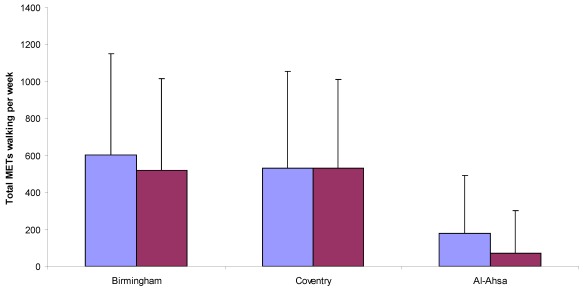
Total METs walking per week of males and females across the three cities.

Univariate ANOVA revealed a highly significant difference in the total walking METs per week of youth across the three cities (F_2,2263_ = 291.50, *p* < 0.001). Bonferroni *post-hoc* analyses revealed that youth from Al-Ahsa were significantly (*p* < 0.001) less active than youth in both Birmingham and Coventry (Figure 2). With respect to gender, univariate ANOVA also revealed significant differences between males and females from Al-Ahsa (F_1,1112_ = 40.435, *p* < 0.001) and Birmingham (F_1,636_ = 4.050, *p* = 0.045). However, there were no significant differences between males and females from Coventry (*p* > 0.05). These findings are illustrated in Figure 2.

### 3.2. Differences in Sedentary Behavior—(TV Viewing + Computer Usage)

Univariate ANOVA revealed that there were no significant differences (*p* > 0.05) in youth sedentary behavior across Birmingham, Coventry and Al-Ahsa. With respect to gender, univariate ANOVA revealed significant differences in sedentary behavior between males (5 hours/day) and females (5.78 hours/day) from Al-Ahsa (F_1,1110_ = 22.106, *p* < 0.001). However, there were no significant differences (*p* > 0.05) between males and females from Birmingham or Coventry (Table 1). A two-way ANOVA revealed that there were no significant interactions between sedentary time, gender and city (*p* > 0.05).

### 3.3. Differences in BMI Classifications

Univariate ANOVA revealed that there were highly significant differences in the BMI of youth across the three cities (F_2,2217_ = 39.613, *p* < 0.001). Bonferroni *post-hoc* analyses revealed that the BMI of youth from Al-Ahsa were significantly higher than youth in Birmingham and Coventry (both, *p* < .001). The mean BMI values for Birmingham, Coventry and Al-Ahsa youth were 22.38, 21.41 and 24.02 (kg/m^2^) respectively. The BMI classifications according to the IOTF are presented in Table 3. These figures indicate that youth from Al-Ahsa have a high prevalence of overweight/obesity (36.4% and 36.6% of males and females respectively). As for gender, univariate ANOVA revealed no significant differences between males and females in each of the three cities. Also, in comparing various age groups, univariate analyses revealed no significant differences in BMI (*p* > 0.05). A two-way ANOVA revealed that there was no significant interaction between BMI score, gender and city (*p* > 0.05).

**Table 3:** BMI classification according to IOTF across the three cities.

City	Gender	N	Body Mass Index (kg/m^2^)			
			Normal	Overweight	Obese	Overweight/obese
**Birmingham**	Males	308	73.4%	20.5%	6.1%	26.6%
	Females	329	73.3%	19.5%	7.2%	26.7%
	Total	637	73.3%	19.9%	6.8%	26.7%
**Coventry**	Males	264	78.5%	14.4%	6.1%	20.5%
	Females	197	82.2%	14.7%	3%	17.8%
	Total	461	80.7%	14.5%	4.8%	19.3%
**Al-Ahsa**	Males	580	63.6%	16.9%	19.5%	36.4%
	Females	538	63.4%	19.5%	17.1%	36.6%
	Total	1,118	63.5%	18.2%	18.3%	36.5%

### 3.4. Associations Between Physical Activity, Sedentary Behavior and BMI

Pearson’s correlation revealed that, in respect of Al-Ahsa city, total PA (METs-min/week) has a significant negative association with BMI (r = −0.180, *p* < 0.001) and with computer-use (r = −0.063, *p* = 0.038). There was also a significant negative association between walking METs per week and BMI (r = −0.112, *p* < 0.001). Furthermore, BMI had a significant positive association with computer-use (r = 0.068, *p* = 0.024) and sedentary behavior (r = 0.078, *p* = 0.010). Also, Pearson’s correlation revealed that, with regard to Birmingham, total PA (METs/week) had significant negative associations with TV viewing (r = −0.103, *p *= 0.010) and sedentary behavior (r = −0.100, *p *= 0.012). However, these relationships which were apparent in Al-Ahsa and Birmingham were not evident in Coventry. 

## 4. Discussion

The UK and SA have both witnessed significant lifestyle changes during the last three decades. Subsequently, physical inactivity, sedentary lifestyle and an ever-increasing rate of obesity have become prevalent in these countries amongst young people and adults [37,38,39]. Despite the multiple health gains associated with a physically active lifestyle, there are high levels of inactivity across the UK, with participation in PA declining significantly with age in both males and females. Boys are generally more active than girls and girls are more likely than boys to reduce their activity levels as they move from childhood to adolescence [5].

The findings of the current study have demonstrated that youth in Al-Ahsa city are significantly less active and have a higher percentage of obesity and sedentary time than those living in Birmingham and Coventry. The low levels of PA amongst youth from Al-Ahsa might be due to lack of availability of sports grounds, parks and facilities that are suitable to engage in physical activities or sport. Moreover, the desert climate of SA is normally not conducive to engagement in PA for a substantial part of the year. Additionally, in general, males seem to report more PA (METs per week) than females across the three cities (Figure 1). The gender differences found in this study seem to concur with the findings from several other studies which indicated that males are generally more physically active than females. For example, across the UK, it has been reported that boys are more likely than girls to be active at almost every age, with an average of 32% of boys and 24% of girls meeting the recommended levels of PA for young people. Furthermore, PA seems to decline with age in both sexes, but more steeply in girls [5]. The current study has revealed that youth in Al-Ahsa reported significantly less total walking (METs/week) than those living in Birmingham or Coventry, with males reporting more walking than females in Al-Ahsa and Birmingham but not Coventry. This indicates that while gender differences were evident across the three cities when total METs per week of all physical activities were analyzed, this was not the case when only total walking METs were concerned. The data evidencing no difference in total walking METs between girls and boys in Coventry is in contrast to the data presented here in relation to Birmingham and Al-Ahsa. This is also contrary to a wide range of studies [23] which have documented higher PA in boys compared to girls. However, the difference in PA from walking between Coventry and Birmingham is not unexpected due to the historical development of the two cities in question. Coventry was extensively bombed during the Second World War and was reconstructed during the 1960s. This has resulted in a built environment which has poor street connectivity, less green space and lower residential density than Birmingham [26,27,40]. These factors have been widely reported to influence walkability in built environments [41,42]. In the case of the present study, the lower residential density, less green space and poorer street connectivity in Coventry compared to Birmingham may have resulted in a ceiling effect for walking METs. Subsequently, walking activity for males in the city of Coventry may be artificially constrained due to the built environment in which they live resulting in similar levels of walking to their female peers and lower levels of walking compared to males from Birmingham.

The weight status classification of youth from different cities indicates that there is a higher percentage of overweight and obese amongst youth from Al-Ahsa compared to those from Birmingham and Coventry. This appears to be the case for both sexes (see Table 3). However, the results indicated that there were no significant differences between males and females with regard to the prevalence of overweight/obesity in each of the three cities when analyzed separately. The current study revealed that the combined prevalence of overweight and obesity amongst the 1,118 youth from Al-Ahsa was 36.5%, with 18.2% overweight and 18.3% obese. A previous study by Amin, Al-Sultan and Ali [43] revealed that the combined prevalence of obesity and overweight was 23.9% (9.7% obese and 14.2% overweight) among Saudi males aged 10–14 years from the Al-Ahsa region. In a cross-sectional, national epidemiological household survey that involved 13,177 participants aged 15 years and over in SA, the prevalence of overweight (BMI 25–30), was higher among males than females (29% *vs.* 27%), while the prevalence of obesity (BMI >30), was higher among females than males (24% *vs.* 16%) [44]. This equates to 45% of males and 51% of females being classified as overweight or obese. Furthermore, a recent study by El Mouzan *et al. *[25] reported that the overall prevalence of overweight was 11.7% and obesity was 15.8% amongst males aged 6 to 18 years, with the highest prevalence of obesity recorded in the capital city of Riyadh (18%). Also, a study on the dietary behavior and lifestyle of Saudi female university students reported an overweight and obesity prevalence of 31.4% and 16.5% respectively. This represents a total of 47.9% of young female adults who were either overweight or obese [45]. Additionally, a recent systematic review paper on obesity in Gulf Co-operation Council States which included 45 studies, reported that the prevalence of overweight and obesity in adults was 25–50% and 13–50% respectively, with a higher prevalence of obesity amongst females [46]. The findings of these studies point out the increasing prevalence of obesity in recent years. These high percentages of overweight and obesity concur with the findings of the current study which indicated a high prevalence amongst both females and males (36.6% and 36.4%, respectively) in SA.

The findings of the current study revealed that the youth from Al-Ahsa appeared to be the least active and had the highest prevalence of overweight/obesity (36.5%) compared to both Birmingham (26.7%) and Coventry (19.3%) youth. The prevalence of obesity amongst Saudi youth seems to be evident across both sexes and exceeds that of other regions of the world. For example, Wang and Lobstein [47] reported a combined prevalence of overweight and obesity of 46.4% in the Americas, 41.7% in Eastern Med, 38.2% in Europe and 22.9% in South East Asia. However, a recent study comparing samples of Kenyan and Canadian children aged 9–13 years from urban and rural environments, reported that none of the rural Kenyan children were overweight or obese; however, 6.8% of the boys and 16.7% of the girls in the urban sample were overweight/obese [48]. Moreover, the prevalence of overweight/obesity amongst the Canadian children ranged from 23% to 28% [48].

The low levels of PA and high percentage of overweight/obese amongst males and females from Al-Ahsa might be related to certain aspects of their lifestyle, dietary habits and environmental factors. For example, using saturated fat in traditional cooking is commonplace in the Gulf countries. A recent study by Washi and Ageib [49] on poor diet quality and food habits of Saudi youth found an increase in dietary intake or energy from fats as well as the fact that rice, bread and meat are regarded as the staple diet and are used in almost every meal. This seems to concur with other studies (on this age group) which found that obese children and adolescents consume significantly more servings of meat, grain products, fast foods, sugar, sweetened drinks and potato chips. These contribute to a higher caloric intake compared to non-obese children and adolescents [50].

The current study found that there is a negative relationship between levels of PA and obesity, with those who report higher levels of PA tending to possess lower BMI. A similar relationship was also evident between PA and computer-usage. This indicates that the more active the individual, the less time spent on computer-usage. Additionally, a significant relationship was evident between weight status and sedentary lifestyle as measured by inactivity; a higher BMI was evident amongst those who reported greater computer-usage time. Also, with regard to youth from Birmingham, total PA METs per week had significant negative associations with TV viewing and sedentary behavior. However, these relationships which were apparent in Al-Ahsa and Birmingham were not evident with youth from Coventry.

Previous research findings have pointed out that the prevalence of obesity amongst youth over the past three decades seems to be increasing in almost all industrialized countries and in several lower-income countries [47]. In societies that have been undergoing rapid socioeconomic transitions (e.g., SA), obesity has increased at an accelerated rate with the prevalence of overweight or obesity in school-age children increasing significantly in several industrialized countries, such as Canada, the United States, Brazil, Greece and the UK [47].

Generally, engagement in PA by young people in SA is not regarded as a desired pursuit (leisure time activity) due to cultural attitudes and beliefs. It is commonly perceived that the pursuit of academic excellence has greater status than PA. Normally, parents encourage their children to engage in educational and spiritual activities rather than leisure time activities. There is a general lack of availability of parks, sports grounds and facilities that are suitable for youth to engage in physical activities or sports. Furthermore, the climate is not conducive to engagement in PA in the outdoors for a substantial part of the year, a problem further compounded by the harsh desert environment and the absence of walkability and appropriate indoor facilities for exercise. This might be part of the reason why youth in Al-Ahsa are less active than those living in Birmingham and Coventry. Also, it is plausible that youth living in Central England have greater availability and access to sporting facilities than those from the Eastern Provence of SA. Moreover, attitudes, societal norms and expectations of communities in SA are generally less amenable towards engagement in sporting activities that require adherence to particular forms of dress than other communities. Normally, youth living in developed countries have better access to sports facilities than those living in developing countries. Those involved in planning policies and designing obesity prevention strategies may use these findings to plan and implement intervention strategies that aim to prevent rather than cure obesity. Emphasizing the hazards of sedentary lifestyle and the importance of healthy nutritional habits to schoolchildren and their parents is paramount in combating the obesity epidemic. Any proposed strategy for tackling sedentary lifestyle and obesity should be multi-disciplinary in nature and target the general population with greater attention to young females. Future interventions could aim to capitalize on the interest of youth in computers/TV by utilizing these technologies to promote desirable habits and encourage more physical activity. An example of this could be through developing active computer games and videos that are culturally relevant and gender specific. Further studies on lifestyle and health habits of youth in other cities of contrasting environment are necessary for a better understanding of environmental and cultural influences.

This study has a number of strengths including the use of appropriate tools for data collection, recruiting representative samples of youth from the three geographical locations that allowed for assessing differences due to gender and age. The study used a standardized protocol and the same instrument for data collection. The PA questionnaire was validated on Saudi youth and was adapted to capture PA of youth in UK. The study provided initial insights into youth PA patterns and weight status in Birmingham, Coventry and Al-Ahsa. This included the contrast in levels of PA, obesity and sedentary activities as well as gender differences. Also, there are a number of limitations to this study. In addition to the inherent limitations of self-report approaches in the assessment of PA and sedentary behavior, the differences in maturation status between youth from the three cities may have affected obesity classification. Therefore, future studies could address these limitations and utilize a mixed approach in data collection through both objective and self-report measures of PA. Also, youth’s perceptions could be recorded through focus groups and interviews to further explore other causes and aspects of their lifestyle and health behaviors.

## 5. Conclusions

The lifestyle and nutritional habits of young people in both developed and developing counties have undergone a major transformation over the past few decades. This is particularly evident in high-income countries such as the UK and SA, where a sedentary lifestyle and an unhealthy diet that is based on high-density processed food offered by a huge range of fast food outlets has contributed to a low level of PA and a high rate of obesity amongst young people. This cross-cultural explorative study investigated the levels of PA, obesity and sedentary activities of youth from three cities in two countries of contrasting culture, lifestyle, geography and climate. The findings of this study demonstrated that gender, and geographical locations seem to influence youth PA levels and obesity. Youth living in two cities in Central England were more physically active than their counterparts living in a city in the Eastern Province of SA. Males were generally more active than females, with females exhibiting higher rates of overweight/obesity. Young males and females from Al-Ahsa reported less PA and recorded higher percentage of overweight and obesity than youth in Birmingham and Coventry. The disparity in PA and weight status between youth from the UK and those from SA could be due to cultural and environmental differences such as the lack of opportunities to exercise, particularly for females, due to societal norms and constraints as well as lifestyle habits.
